# Effectiveness of injury prevention programmes in female team sports: a systematic review and meta-analysis of hierarchical injury outcomes, training dose–response relationships, and adherence

**DOI:** 10.3389/fpubh.2026.1950998

**Published:** 2026-09-15

**Authors:** Jinfa Gu, Yan Li, Hengzhi Deng, Yanan Qi, Lin Xiao, Ruohan Zhang

**Affiliations:** 1 Institute of Physical Education, Jiangsu Normal University, Xuzhou, Jiangsu, China; 2 School of Physical Education, Henan Polytechnic University, Jiaozuo, Henan, China; 3 Department of Pharmacy, Ezhou Central Hospital, Ezhou, Hubei, China; 4 Faculty of Sports and Exercise Science, University of Malaya, Kuala Lumpur, Malaysia; 5 Department of Nursing and Rehabilitation, Xi'an Jiaotong University City College, Xi'an, Shaanxi, China; 6 School of Physical Education and Health, Zhaoqing University, Zhaoqing, Guangdong, China

**Keywords:** adherence, dose–response, female athletes, injury prevention, neuromuscular training

## Abstract

**Objective:**

To quantify the effectiveness of exercise-based injury prevention programmes (IPPs) in female team sports across overall, lower-limb, and site-specific injury outcomes, and to examine training dose and adherence as potential effect modifiers.

**Methods:**

Randomised controlled trials evaluating exercise-based IPPs in female team-sport athletes were synthesised. Risk ratios (RRs) were pooled using random-effects models. Subgroup analyses examined training frequency, session duration, weekly training dose, and adherence, while meta-regression was used to examine study-level associations with training dose and adherence.

**Results:**

Thirteen unique trials involving 13,675 athletes were included. IPPs reduced overall injury risk by 35% (RR = 0.65, 95% CI: 0.53–0.79), with a similar reduction in lower-limb injuries (RR = 0.67, 95% CI: 0.53–0.86). Significant reductions were also observed for anterior cruciate ligament injuries (RR = 0.62, 95% CI: 0.43–0.89) and ankle injuries (RR = 0.59, 95% CI: 0.42–0.84). Exploratory subgroup analyses suggested larger effects at 4–5 sessions/week (RR = 0.47) and 21–30 min/session (RR = 0.42). Programmes providing 51–120 min/week were associated with greater risk reduction than those providing 1–50 min/week (RR = 0.48 vs. 0.80). Trials with adherence ≥75% showed a clear protective effect (RR = 0.55), whereas those with lower adherence showed little evidence of benefit (RR = 0.93). The certainty of evidence for the primary overall-injury outcome was low.

**Conclusion:**

Exercise-based neuromuscular IPPs appear to reduce overall and lower-limb injury risk in female team sports, with protective effects observed for ACL and ankle injuries and the most consistent estimate for ACL injury. Greater weekly exposure was associated with larger risk reductions at the study level, whereas the continuous adherence meta-regression was not significant. These patterns should be interpreted as exploratory implementation signals rather than definitive thresholds.

**Systematic review registration:**

The systematic review was prospectively registered in PROSPERO (CRD420251236606). URL: https://www.crd.york.ac.uk/PROSPERO/view/CRD420251236606

## Introduction

Musculoskeletal injuries among female team-sport athletes represent a major public health concern, with substantial implications for sports participation, performance, and long-term health (1–3). Over the longer term, severe or recurrent injuries may precipitate premature retirement from sport, persistent symptoms, and increased healthcare utilisation (4, 5), and markedly elevate the risk of post-traumatic osteoarthritis. These consequences underscore the importance of developing and implementing effective, evidence-based injury-prevention strategies specifically tailored to female athletes (3, 6, 7).

Over the past two decades, a variety of exercise-based injury-prevention programmes (IPPs) have been developed and widely implemented in team sports such as football, basketball, handball, and volleyball. These programmes are typically neuromuscular and combine strength training, balance and proprioceptive exercises, plyometrics, core stability work, and drills targeting running, cutting, and landing (2, 8). The underlying rationale is that improving dynamic lower-limb joint stability and optimising the biomechanics of key high-risk movements, such as jump-landing, deceleration, and changes of direction, reduces mechanical loading on vulnerable structures and thereby lowers the risk of injury (9–11). Multiple randomised controlled trials and several meta-analyses suggest that IPPs may reduce overall and lower-limb injury rates (12, 13). However, effect sizes and consistency vary considerably between studies, and it remains unclear which populations, delivery formats, and training doses these programmes achieve the most significant preventive benefit (1, 13).

Epidemiological data in female team sports indicate that lower-limb injuries account for a large proportion of time-loss injuries (7, 14), with knee, ankle, and anterior cruciate ligament (ACL) injuries being highly prevalent and strongly associated with long-term functional limitations and shortened athletic careers (1, 6, 15).

Several systematic reviews and meta-analyses have summarised the preventive effects of IPPs, focusing on specific programmes, such as FIFA 11 + and PEP (2, 5, 6), particular injury types, such as ACL and ankle sprains, or single sports, predominantly football (4, 8). However, existing reviews are typically constrained to single sports, injury types, or narrow populations, providing limited insight into how effects vary across anatomical sites, injury contexts, and implementation characteristics (1, 4).

Despite these advances, several critical questions remain inadequately addressed. First, few reviews have specifically quantified these outcomes within female team-sport randomised trials, particularly alongside dose–response and adherence analyses. Second, the effectiveness of IPPs in different injury contexts (training versus match, acute, severe, non-contact) has not been comprehensively quantified. Third, few reviews have combined subgroup analyses with meta-regression to examine dose–response relationships systematically between training dose and injury-risk reduction, or to quantify the impact of adherence (12). Whether the type of sport, age, competitive level, programme format, and delivery mode modify IPP effects also remains uncertain.

The FAIR IOC consensus provides broad recommendations for injury prevention in female athletes. Complementing this guidance, the present review synthesises randomised evidence specifically from female team sports, integrating injury outcomes, training dose, and adherence within a single analysis (16).

Against this background, a systematic review and meta-analysis of randomised controlled trials in female team-sport athletes was conducted to evaluate the impact of exercise-based IPPs on sports injury risk. The primary objective was to determine whether IPPs reduce the overall incidence of sports injuries compared with usual training. Secondary objectives were to: (1) assess IPP effects on lower-limb and site-specific injuries (knee, ankle, ACL, hamstring, and hip/groin) within a conceptual outcome hierarchy; (2) compare effect patterns across injury contexts (training versus match, acute, severe, non-contact); (3) examine study-level associations between weekly training dose and injury risk using subgroup analyses and meta-regression; (4) examine adherence as a moderator of effectiveness; and (5) explore whether sport type, age, competitive level, and programme format modify preventive effects. The *a priori* hypothesis was that IPPs would reduce overall and lower-limb injury risk, with greater reductions associated with higher training doses and higher adherence.

## Methods

### Methodology and literature search strategy

The protocol for this systematic review and meta-analysis was prospectively registered in PROSPERO (CRD420251236606), and the review was conducted in accordance with the PRISMA 2020 statement (17). Five electronic databases (PubMed, Web of Science, Scopus, Embase, and the Cochrane Library) were searched from their inception through August 21, 2026. The search strategy was structured around consistent core concepts for injury, female athletes, team sports, exercise-based IPPs, and randomised trials, with database-specific controlled vocabulary, free-text terms, field tags, and limits applied as appropriate. The full database-specific search strategies are provided in Supplementary Table S1. Reference lists of eligible articles and relevant reviews were manually screened to identify additional reports. When eligibility could not be confirmed from the reported data, the authors were contacted for additional information. Two reviewers independently screened titles/abstracts and full-text records. Disagreements were resolved by discussion and, where necessary, consultation with a third reviewer.

### Inclusion and exclusion criteria

Randomised controlled trials were eligible for inclusion if they evaluated structured, exercise-based IPPs in female team-sport athletes. Eligible IPPs were defined as structured, exercise-based programmes intended to prevent sports injuries and incorporating one or more preventive exercise components, including strength, balance/proprioception, plyometrics, landing or movement-control exercises, and core stability. Eligible interventions could be single-component or multicomponent and could be delivered as part of a warm-up or structured conditioning programme. Established programmes such as FIFA 11+, PEP, and Knee Control, as well as other interventions meeting this operational definition, were eligible. Although programme content and delivery format varied, interventions meeting this operational definition were synthesised within the same exercise-based IPP framework because they met the same operational eligibility definition. Differences in programme format were explored in subgroup analyses, and random-effects models were used throughout the meta-analysis. The target population comprised female athletes participating in organised team sports, such as football/soccer, basketball, and floorball, and studies were included only when data for female participants could be extracted separately. The primary outcome was the overall incidence of sports injuries. Secondary outcomes included lower-limb injuries, site-specific injuries of the knee, ankle, ACL, hip/groin, and hamstring, and context- and type-specific outcomes, including training- versus match-related injuries and acute, severe, and non-contact injuries.

Overall injuries were analysed as the broadest trial-level outcome, whereas lower-limb and site-specific injuries were analysed as secondary outcomes as defined in the original studies. Here, “hierarchical” refers to the conceptual organization of outcomes from broader to more specific injury categories; each outcome category was meta-analysed separately.

Studies were excluded if they used non-randomised or quasi-experimental intervention designs, including controlled or uncontrolled before–and–after studies; if the intervention did not consist of a structured, exercise-based IPP, such as usual warm-up routines or regular training without a defined preventive component; if they involved non-team-sport athletes or did not report separable data for female athletes; if they lacked quantitative injury outcomes; or if they were not peer-reviewed, full-text articles.

### Data extraction

Two independent reviewers extracted data into a standardised Excel spreadsheet using a predefined extraction form. Extracted variables included bibliographic and study characteristics (first author, year, country, and study design), athlete information (age, sport, and competitive level), intervention characteristics (training frequency, session duration, intervention duration, adherence, and type of neuromuscular training programme), sample size analysed for intervention and control groups, and the injury outcomes reported. Adherence was recorded as the percentage of prescribed sessions that were completed. When multiple reports described the same trial, the reports were linked and treated as a single study. The primary trial report was used for the intervention-effect estimate, whereas companion reports were used to obtain additional information on intervention characteristics, including adherence, when relevant. Each trial contributed only one intervention-effect estimate to any quantitative analysis. Any discrepancies were resolved by consensus or, if required, by a third independent reviewer.

### Quality assessment

Risk of bias for the primary overall-injury outcome was assessed using the Cochrane Risk of Bias 2 (RoB 2) tool (18). The standard RoB 2 version was applied to individually randomised trials, whereas the cluster-randomised trial version was applied when allocation occurred at the team, school, club, or other cluster level. The RoB 2 CRT version was applied when sufficient information on cluster-level recruitment and allocation procedures was available. Assessments targeted the effect of assignment to intervention. Two reviewers independently completed the assessments, with disagreements resolved through discussion or consultation with a third reviewer.

### Certainty of the evidence

The certainty of evidence for the primary overall-injury outcome was assessed using the GRADE approach, considering risk of bias, inconsistency, indirectness, imprecision, and publication bias, and was categorised as high, moderate, low, or very low (19).

### Data analysis

Injury risk was defined as the proportion of athletes who sustained at least one injury during the study follow-up period, as reported in the original trials. Risk Ratios (RRs) with 95% confidence intervals (CIs) were used to quantify intervention effects. Study-specific log RRs and corresponding standard errors (SEs) were pooled using an inverse-variance random-effects model. No additional design-effect correction was applied to cluster-randomised trials because intracluster correlation coefficients and sufficient cluster-level information were not consistently available. Statistical heterogeneity was assessed using the Cochrane Q test and quantified by the I^2^ statistic (20). The 95% prediction interval was calculated to estimate the range of true effects in future studies (21).

Subgroup analyses were performed to examine the influence of intervention dose characteristics (training frequency, session duration, intervention period, and weekly training dose), participant factors (age group, sport type, and competitive level), intervention type, and adherence on the preventive effectiveness of IPPs. For consistency, adherence was categorised according to completion rate as low (<75%) and high (≥75%). The 75% threshold was selected as a pragmatic study-level cut-point approximating the median adherence reported across included trials. Subgroup differences were tested using χ^2^ tests for interaction. Statistical significance was set at *p* < 0.05 for all analyses, including main effects, subgroup interactions, and meta-regression coefficients, unless otherwise specified.

Separate univariable random-effects meta-regressions were conducted to examine study-level associations of weekly training dose (minutes per week) and adherence percentage with injury risk. Moderator coefficients with 95% confidence intervals, residual τ^2^, and the proportion of between-study heterogeneity explained by the moderator (R^2^) were reported. Both categorical subgroup comparisons and continuous linear meta-regression models were used to examine these moderators.

Sensitivity analyses were conducted to test the robustness of findings, including a leave-one-out procedure, re-estimation under a fixed-effects model, exclusion of studies with extreme effect estimates (minimum and maximum RR values), removal of small-sample studies (n < 200).

Potential small-study effects and funnel-plot asymmetry were examined using funnel plots, Egger’s regression test (22), and Begg’s rank correlation test (applied when ≥10 studies were available) (23). The trim-and-fill method was additionally performed as a sensitivity analysis to explore the potential influence of funnel-plot asymmetry on the pooled estimate (24).

All analyses were performed in R (version 4.4.3) using the meta and metafor packages within RStudio. Forest and funnel plots were generated in R (25).

## Results

### Literature search results

Database searching identified 1,584 records (Figure 1). After removal of 587 duplicate records, 997 records were screened, of which 948 were excluded. Forty-nine reports were sought for retrieval, and four could not be retrieved; therefore, 45 reports were assessed for eligibility. Of these, 34 reports were excluded after full-text assessment: no assessment of injury (*n* = 9), insufficient data (*n* = 9), ineligible study type (*n* = 11), or no athletes studied (*n* = 5). Citation searching identified three additional reports, all of which were retrieved, assessed for eligibility, and included. Overall, 14 reports corresponding to 13 unique randomised controlled trials were included in the review. The 13 unique trials contributed intervention-effect estimates to the meta-analysis.

**Figure 1:**
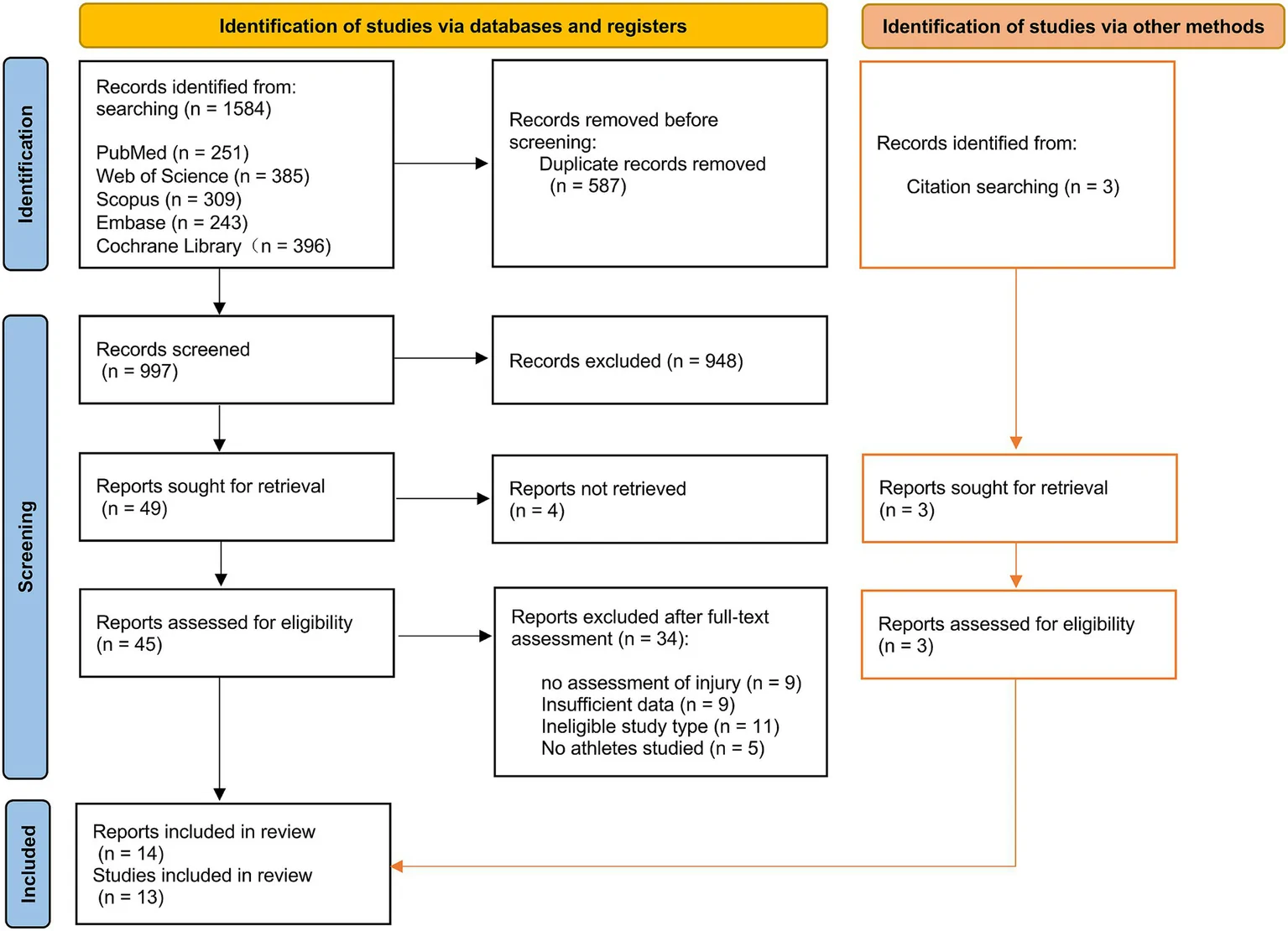
PRISMA 2020 flow diagram showing the identification and selection of 14 eligible reports corresponding to 13 unique randomised controlled trials.

### Characteristics of included studies

Fourteen eligible reports described 13 unique randomised controlled trials (26–39), enrolling 13,675 female athletes with ages ranging from 7 to 24 years, at amateur (9 trials, 69%) and elite (4 trials, 31%) competitive levels. Hägglund et al. (39) was a companion report of the Waldén trial and contributed supplementary adherence information but no additional intervention-effect estimate. The trials were conducted across 10 countries, including one multinational trial involving Switzerland, Germany, the Czech Republic, and the Netherlands.

Football/soccer was the predominant sport, examined in 11 trials (84.6%, 11/13), either exclusively (*n* = 9) or in mixed-sport samples including basketball and/or volleyball (*n* = 2). Single-sport studies in basketball (*n* = 1) and floorball (*n* = 1) comprised the remainder. Analysed sample sizes ranged from 140 to 4,564 participants per trial (median: 457).

Interventions were delivered 1–5 times weekly for 10–30 min over 7–44 weeks. Adherence data were available for 10 trials (77%) and ranged from approximately 52% to 88%. Intervention protocols comprised FIFA-based neuromuscular training programmes (*n* = 4, 31%) and non-FIFA neuromuscular programmes (*n* = 9, 69%) emphasising strength, balance, plyometrics, landing mechanics, core stability, and sport-specific movement patterns. Control groups continued their usual warm-up routines.

Outcome measures encompassed overall injury incidence (*n* = 13) and anatomical site-specific injuries: lower-limb (*n* = 9), knee (*n* = 12), ankle (*n* = 9), anterior cruciate ligament (*n* = 6), hip/groin (*n* = 2), and hamstring (*n* = 2). Study characteristics and intervention protocols are summarised in Table 1.

**Table 1:** Characteristics of included studies and intervention protocols.

First author, year	Age (years)	Country	Athlete population and level	Intervention characteristics (training frequency, session duration, intervention duration, adherence, intervention type)	Sample size analysed, *n* (IG/CG)*	Injury outcomes contributing data to the present meta-analysis
Bonato et al. (2018) (26)	20 ± 2 y	Italy	Elite women’s basketball players; level: elite	Training frequency: 4 sessions/week; session duration: 30 min; intervention duration: 34 weeks; adherence: 81%; intervention type: bodyweight neuromuscular training (NMT) warm-up	160 (86/74)	Overall injuries; lower-limb injuries; knee injuries; ankle injuries
Emery et al. (2010) (27)	13–18 y	Canada	Youth indoor football (soccer), U13–U18; level: amateur	Training frequency: 3 sessions/week; session duration: 15 min; intervention duration: NR; adherence: NR; intervention type: neuromuscular training (NMT) plus home-based balance training	412 (161/251)	Overall injuries; knee injuries; ankle injuries
Foss et al. (2018) (28)	14 ± 1.7 y	USA	High school and middle school basketball, football (soccer), and volleyball; level: amateur	Training frequency: 2–3 sessions/week; session duration: 20–25 min; intervention duration: 39 weeks; adherence: 88%; intervention type: core-focused neuromuscular training (NMT)	474 (259/215)	Overall injuries; lower-limb injuries; ACL injuries; knee injuries; ankle injuries
Gilchrist et al. (2008) (29)	18–22 y	USA	Women’s football (soccer), NCAA Division I; level: elite	Training frequency: 2–3 sessions/week; session duration: 20 min; intervention duration: 14 weeks; adherence: 72%; intervention type: PEP programme	1,435 (583/852)	Overall injuries; lower-limb injuries; ACL injuries; knee injuries; training injuries; match injuries
Heidt et al. (2000) (30)	14–18 y	USA	High-school girls’ football (soccer); level: amateur	Training frequency: 3 sessions/week; session duration: NR; intervention duration: 7 weeks; adherence: NR; intervention type: neuromuscular training (NMT)	300 (42/258)	Overall injuries; ACL injuries; knee injuries; ankle injuries
LaBella et al. (2011) (31)	16.2 ± 1.5 y	USA	High-school girls’ football (soccer) and basketball; level: amateur	Training frequency: 2 sessions/week; session duration: 20 min; intervention duration: 20 weeks; adherence: 80%; intervention type: neuromuscular warm-up programme	1,492 (737/755)	Overall injuries; lower-limb injuries; ACL injuries; knee injuries; ankle injuries; acute injuries
Pasanen et al. (2008) (32)	24 y	Finland	Women’s floorball, elite to 2nd division; level: elite	Training frequency: 2–3 sessions/week; session duration: 20–30 min; intervention duration: 26 weeks; adherence: ≈75%; intervention type: neuromuscular training (NMT) programme	457 (256/201)	Overall injuries; lower-limb injuries; knee injuries; ankle injuries; non-contact injuries
Rössler et al. (2018) (33)	7–13 y	Switzerland, Germany, Czech Republic, Netherlands	Community football (soccer; U9, U11, U13); level: amateur	Training frequency: ≥2 sessions/week; session duration: 15–20 min; intervention duration: 44 weeks; adherence: NR; intervention type: “11 + Kids” neuromuscular warm-up	171 (103/68)	Overall injuries; knee injuries
Soligard et al. (2008) (35)	13–17 y	Norway	Regional district youth girls’ football (soccer); level: amateur	Training frequency: 2–5 sessions/week; session duration: 20 min; intervention duration: 34 weeks; adherence: 77%; intervention type: FIFA 11 + programme	1,892 (1,055/837)	Overall injuries; lower-limb injuries; knee injuries; ankle injuries; hip/groin injuries; hamstring injuries; acute injuries
Steffen et al. (2008) (38)	13–17 y	Norway	U17 regional district girls’ football (soccer); level: amateur	Training frequency: 1 session/week; session duration: 15 min; intervention duration: 34 weeks; adherence: 52%; intervention type: FIFA 11 programme	2,020 (1,073/947)	Overall injuries; lower-limb injuries; knee injuries; ankle injuries; hip/groin injuries; training injuries; match injuries; acute injuries; non-contact injuries
Steffen et al. (2013) (37)	13–18 y	Canada	Youth female football (soccer), U14–U18; level: amateur (Tiers 1–3)	Training frequency: 2–3 sessions/week; session duration: 15–20 min; intervention duration: 19 weeks; adherence: 79.5%; intervention type: FIFA 11 + programme	158 (78/80)	Overall injuries; lower-limb injuries; knee injuries
Söderman et al. (2000) (34)	20.4 ± 4.6 y	Sweden	Women’s football (soccer), Swedish 2nd–3rd divisions (senior); level: Elite	Training frequency: 3 sessions/week; session duration: 10–15 min; intervention duration: 30 weeks; adherence: 68%; intervention type: balance board training	140 (62/78)	Overall injuries; lower-limb injuries; ACL injuries; ankle injuries; hamstring injuries
Waldén et al. (2012)ᵃ (36)	14.0 ± 1.2 y	Sweden	Adolescent female club football (soccer; U13–U18); level: amateur	Training frequency: 2 sessions/week; session duration: ≈15 min; intervention duration: 28 weeks; team-level adherence: 79%ᵃ; intervention type: neuromuscular training programme (Knee Control)	4,564 (2,479/2,085)	Overall injuries; ACL injuries; knee injuries; acute injuries; severe injuries

Age is reported as mean ± SD or range, according to the original articles.

Intervention characteristics refer to the neuromuscular warm-up or training programme used in the intervention group (training frequency, session duration, intervention duration and adherence).

Injury outcomes indicate outcomes that contributed data to the present meta-analysis, using definitions reported in the original studies.

^*^Sample size refers only to female athletes in both intervention and control groups.

ᵃWaldén et al. (36) was the primary trial report and provided the intervention-effect data. The team-level adherence value of 79% was obtained from the companion report by Hägglund et al. (39). Both reports describe the same trial and were treated as one study.

IG, intervention group; CG, control group; NMT, neuromuscular training; ACL, anterior cruciate ligament; NCAA, National Collegiate Athletic Association; NR, not reported; PEP, Prevent injury and Enhance Performance.

### Risk of bias assessment

Risk of bias assessment indicated that all 13 trials raised some concerns, with none judged at low or high risk of bias. Twelve cluster-randomised trials were assessed using the RoB 2 CRT tool. Concerns were mainly related to the randomisation process, the timing of identification and recruitment of participants in relation to cluster allocation (D1b), and deviations from intended interventions. The individually randomised trial by Heidt et al. (30) was assessed using the standard RoB 2 tool and was also judged as raising some concerns overall. Outcome measurement and selection of the reported result were generally judged at low risk of bias (see Figure 2).

**Figure 2:**
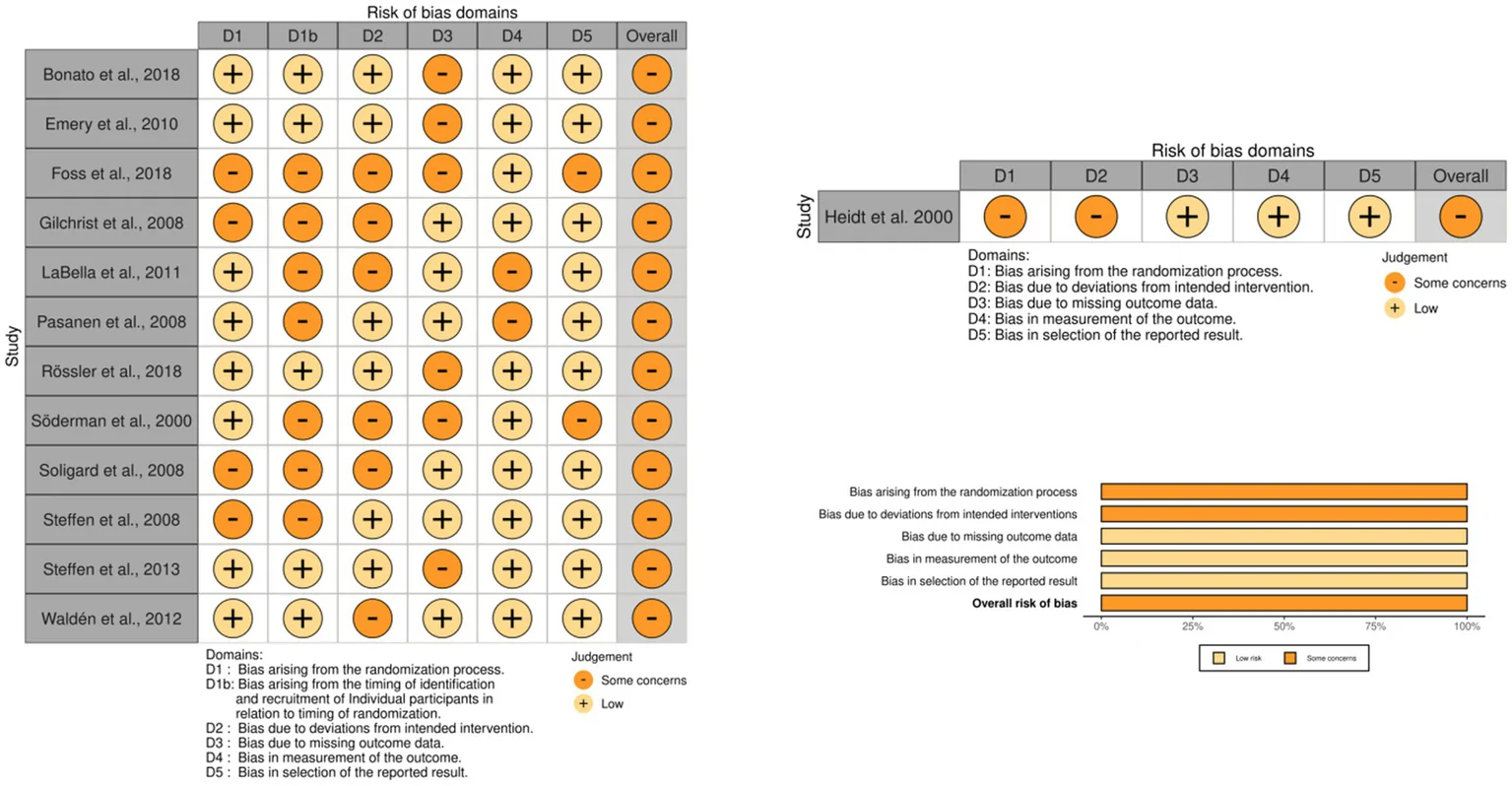
Risk of bias assessment using the Cochrane RoB 2 tool and RoB 2 tool for cluster-randomised trials (RoB 2 CRT).

### Evaluation of meta-analysis results

#### Overall effect test

Pooled results from 13 unique randomised controlled trials (*n* = 13,675) showed that IPPs significantly reduced overall sports injury risk (RR = 0.65, 95% CI: 0.53–0.79; *p* < 0.0001). Substantial between-study heterogeneity was observed (I^2^ = 78%; τ^2^ = 0.086), and the 95% prediction interval (0.33–1.27) suggested that true effects in future studies may vary in magnitude and could include no effect (see Figure 3).

**Figure 3:**
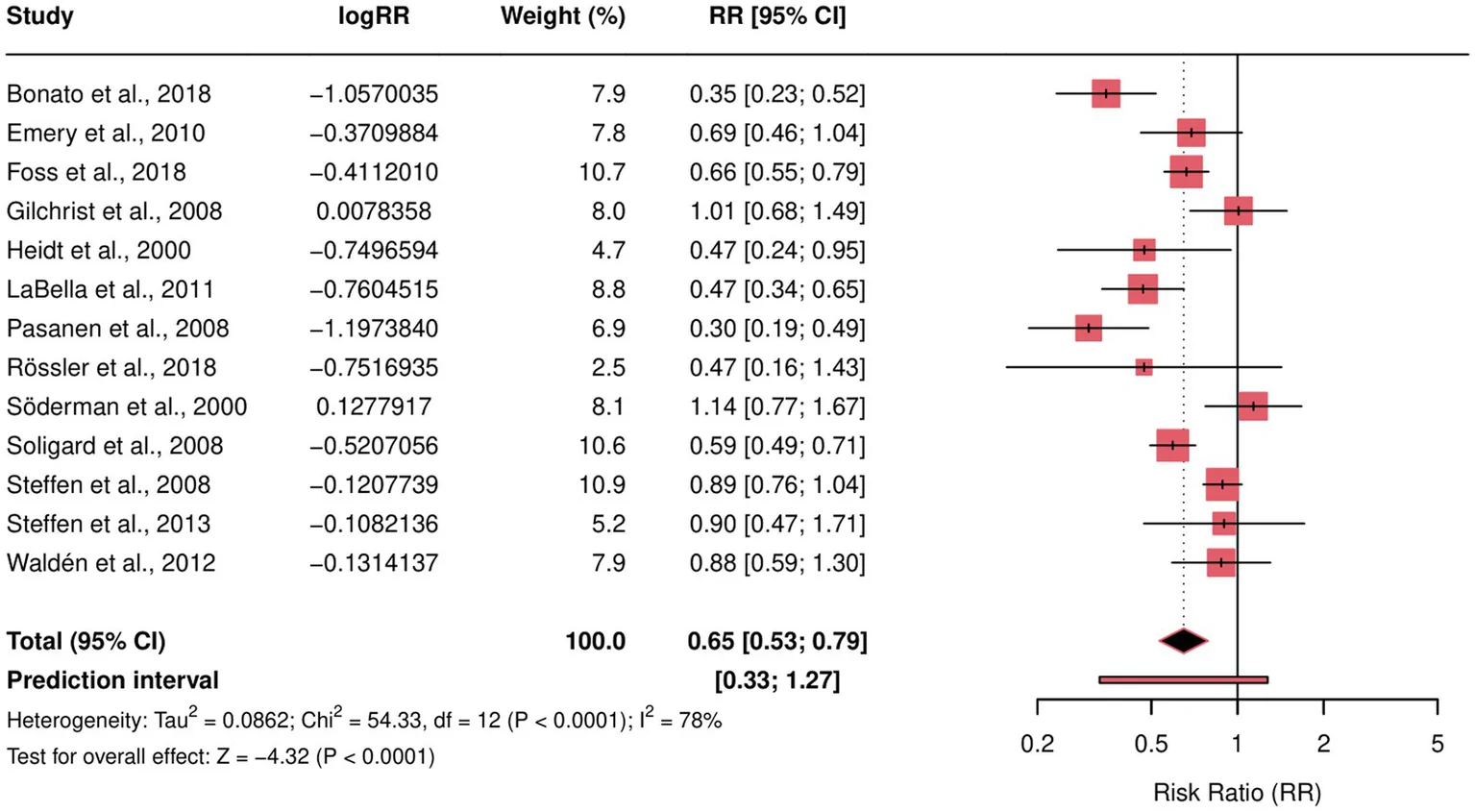
Forest plot of the effects of injury-prevention programmes (IPPs) on overall sports injury risk.

#### Site-specific and context-specific preventive effects

IPPs reduced the risk of lower-limb injuries (RR = 0.67, 95% CI: 0.53–0.86; *p* = 0.002), with protective effects across several anatomical sites (Figure 4A). The effect for anterior cruciate ligament (ACL) injuries was significant and consistent (RR = 0.62, 95% CI: 0.43–0.89; I^2^ = 0%). Significant risk reductions were also observed for ankle (RR = 0.59, 95% CI: 0.42–0.84) and knee injuries (RR = 0.76, 95% CI: 0.59–0.97). By contrast, effects for hip/groin (RR = 0.59; *p* = 0.21) and hamstring injuries (RR = 0.39; *p* = 0.06) did not reach statistical significance, likely reflecting the small number of studies contributing to these outcomes (both *k* = 2) and the resulting limited statistical power.

**Figure 4:**
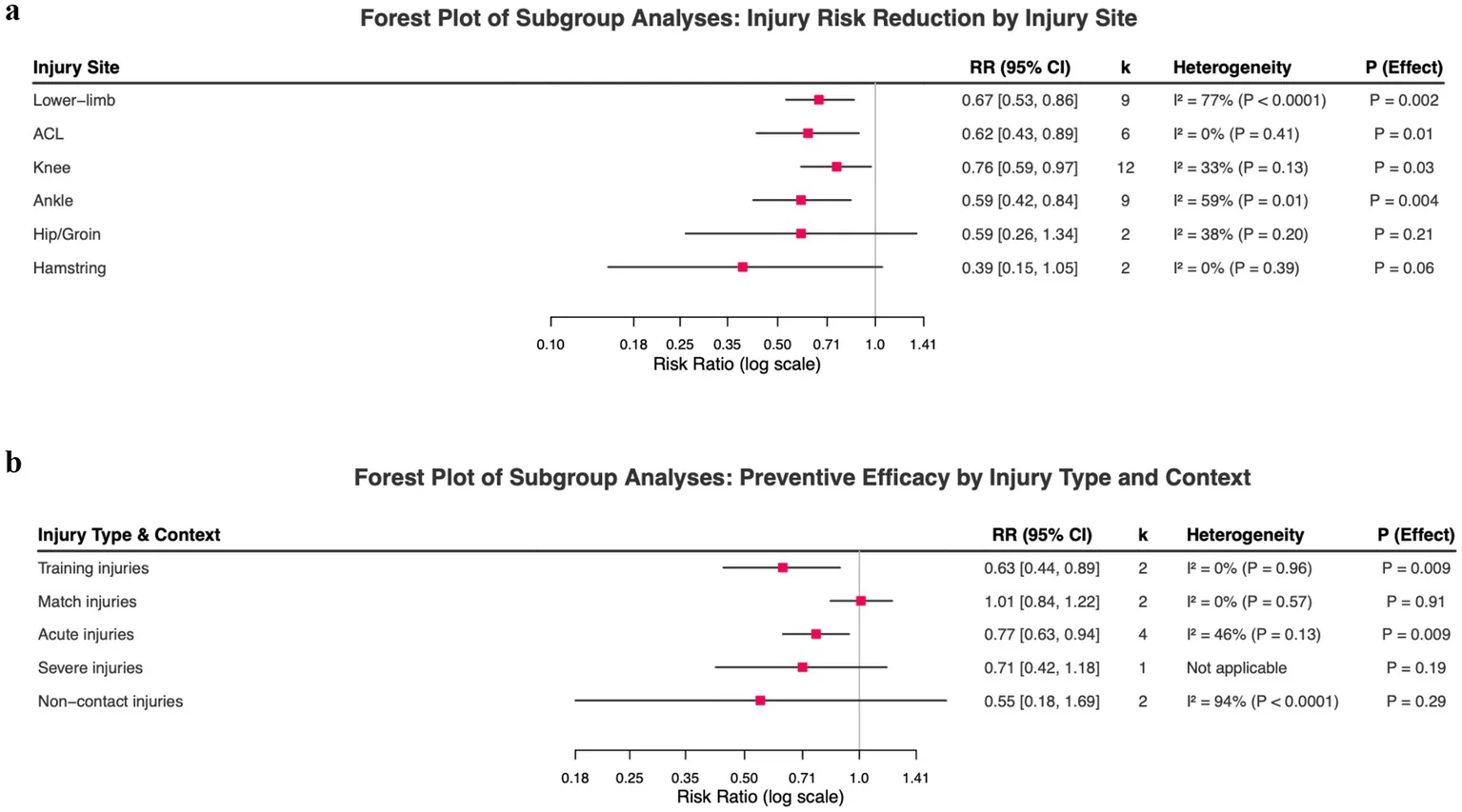
Stratified meta-analyses (forest plots) of the effects of IPPs on lower-limb injury risk: **(a)** By anatomical region and **(b)** By context (training vs. match) and injury type (acute, severe, non-contact).

With respect to injury context and type (Figure 4B), IPPs significantly reduced injury risk during training (RR = 0.63, 95% CI: 0.44–0.89), whereas no clear effect was detected for match-related injuries (RR = 1.01, 95% CI: 0.84–1.22). IPPs were also associated with a reduction in acute injuries (RR = 0.77, 95% CI: 0.63–0.94). The severe-injury estimate was not statistically significant (RR = 0.71, 95% CI: 0.42–1.18; *p* = 0.19; *k* = 1). Although the pooled point estimate for non-contact injuries suggested a protective effect (RR = 0.55), this result did not reach statistical significance in the context of very high heterogeneity (I^2^ = 94%) and a limited number of contributing studies (*k* = 2).

#### Subgroup analyses by sport, training dose, and intervention characteristics

Subgroup estimates varied by sport type and some training-dose parameters (Supplementary Figures S1, S2).

In sport-stratified analyses, between-group differences were significant (P for interaction < 0.0001). Non-soccer sports (*k* = 2; RR = 0.33, *p* < 0.00001) and mixed-sport interventions (*k* = 2; RR = 0.57, *p* = 0.002) showed clear risk reductions, and soccer-only programmes (*k* = 9) were also protective (RR = 0.79, *p* = 0.02), although with a smaller effect size. Both non-soccer and mixed-sport subgroups comprised only two trials, and these results should therefore be interpreted with caution.

For training dose, weekly frequency showed differential effects (P for interaction = 0.02). Programmes delivered two to three times per week (RR = 0.67, *p* = 0.001) and four to five times per week (RR = 0.47, *p* = 0.004) significantly reduced injury risk, whereas once-weekly training did not (*p* = 0.13; one trial). Session-duration differences were borderline (P for interaction = 0.06): interventions lasting 21–30 min (RR = 0.42, *p* = 0.003) and 11–20 min (RR = 0.76, *p* = 0.01) both showed protective effects. Intervention period showed a similar pattern (P for interaction = 0.03); programmes lasting 4–6 months (RR = 0.48, *p* = 0.0002) and 7–12 months (RR = 0.69, *p* = 0.003) were effective, whereas 1–3-month interventions were not (RR = 1.01, *p* = 0.97; *k* = 1).

No significant between-group differences were observed for programme type, age group, or competitive level (P for interaction = 0.42, 0.72, and 0.72, respectively), although within-subgroup effectiveness varied. Significant reductions were observed in adolescents (RR = 0.67, *p* < 0.00001) and amateur athletes (RR = 0.67, *p* < 0.00001), but not in adults (RR = 0.59, *p* = 0.13) or elite athletes (RR = 0.59, *p* = 0.13); both latter subgroups comprised four studies with substantial heterogeneity.

Non-FIFA neuromuscular programmes significantly reduced injury risk (RR = 0.62, 95% CI: 0.47–0.81; *p* = 0.0005), whereas the FIFA-based subgroup was at the threshold of statistical significance (RR = 0.73, 95% CI: 0.53–1.00; *p* = 0.05). There was no evidence of a between-group difference (P for interaction = 0.42). These subgroup analyses were exploratory, and substantial residual heterogeneity remained in several subgroups; therefore, significant interaction tests should not be interpreted as showing that the examined moderators fully explain between-study heterogeneity.

#### Meta-regression analyses (weekly dose–response and adherence)

For weekly training dose, both approaches suggested an inverse study-level association with injury risk. In subgroup analyses, programmes providing 51–120 min/week were associated with a larger pooled risk reduction (RR = 0.48, 95% CI: 0.35–0.66) than programmes providing 1–50 min/week (RR = 0.80, 95% CI: 0.65–1.00), with a significant between-subgroup interaction (P for interaction = 0.009). The low-dose estimate was borderline (*p* = 0.05), whereas the high-dose estimate was clearly significant (*p* < 0.00001). In the corresponding univariable meta-regression (*k* = 12), weekly training minutes were inversely associated with injury risk at the study level (*β* = −0.0096, 95% CI: −0.0157 to −0.0036; *p* = 0.002), with residual τ^2^ = 0.0408 and R^2^ = 53.4%.

Categorical adherence analyses showed a significant reduction in trials with high adherence (≥75%; *k* = 7; RR = 0.55, 95% CI: 0.43–0.70; *p* < 0.001), whereas trials with lower adherence (<75%; *k* = 3) showed little evidence of benefit (RR = 0.93, 95% CI: 0.81–1.06; *p* = 0.28). The between-subgroup interaction was statistically significant (*p* = 0.002). By contrast, the univariable continuous meta-regression (*k* = 10) did not show a statistically significant study-level association between adherence percentage and injury risk (β = −0.0166, 95% CI: −0.0385 to 0.0052; *p* = 0.136), with residual τ^2^ = 0.0945 and R^2^ = 2.3% (see Figure 5).

**Figure 5:**
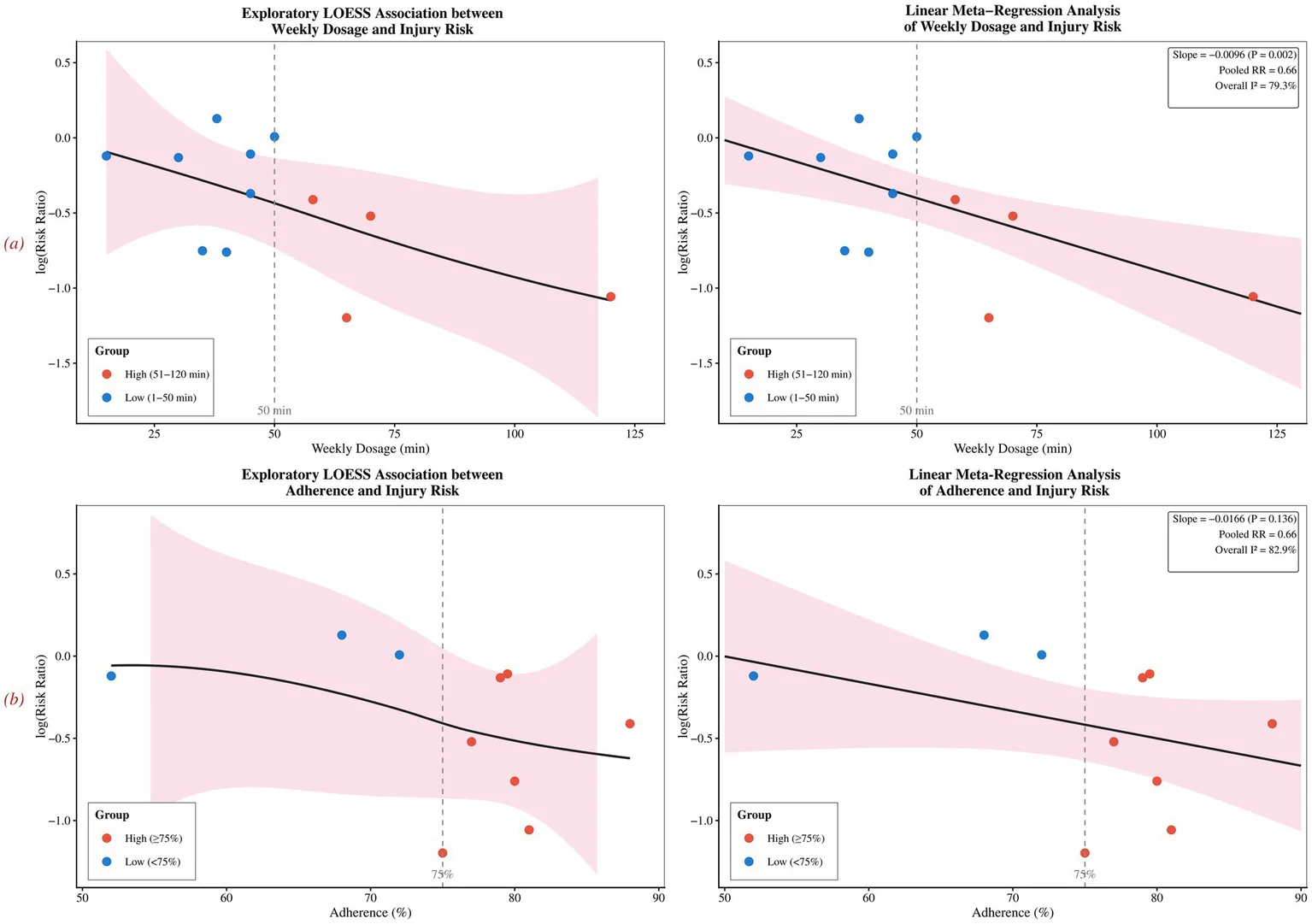
Associations of weekly training dose and adherence with injury risk: **(a)** weekly training dose; **(b)** adherence.

#### Sensitivity analyses of the pooled effect

Leave-one-out analyses yielded pooled RRs ranging from 0.62 to 0.69, and every estimate remained statistically significant. A fixed-effect model produced a similar estimate (RR = 0.69, 95% CI: 0.64–0.75). Excluding the study with the smallest RR yielded RR = 0.69 (95% CI: 0.57–0.83) (32), whereas excluding the study with the largest RR yielded RR = 0.62 (95% CI: 0.51–0.75) (34). Exclusion of trials with analysed samples below 200 participants also produced a similar random-effects estimate (RR = 0.65, 95% CI: 0.53–0.79).

#### Assessment of small-study effects and funnel-plot asymmetry

Egger’s test (*p* = 0.3955) and Begg’s test (*p* = 0.2519) did not detect statistically significant small-study effects. However, given the limited number of included trials, these tests may have had limited power to detect funnel-plot asymmetry. In the trim-and-fill sensitivity analysis, three potentially missing studies were imputed on the right side of the funnel plot, and the pooled estimate was attenuated from RR = 0.648 (95% CI: 0.532–0.789) to RR = 0.767 (95% CI: 0.618–0.952; *p* = 0.016). The trim-and-fill analysis was therefore interpreted as a sensitivity analysis, and the resulting estimate was not regarded as a definitive bias-corrected estimate (see Figure 6).

**Figure 6:**
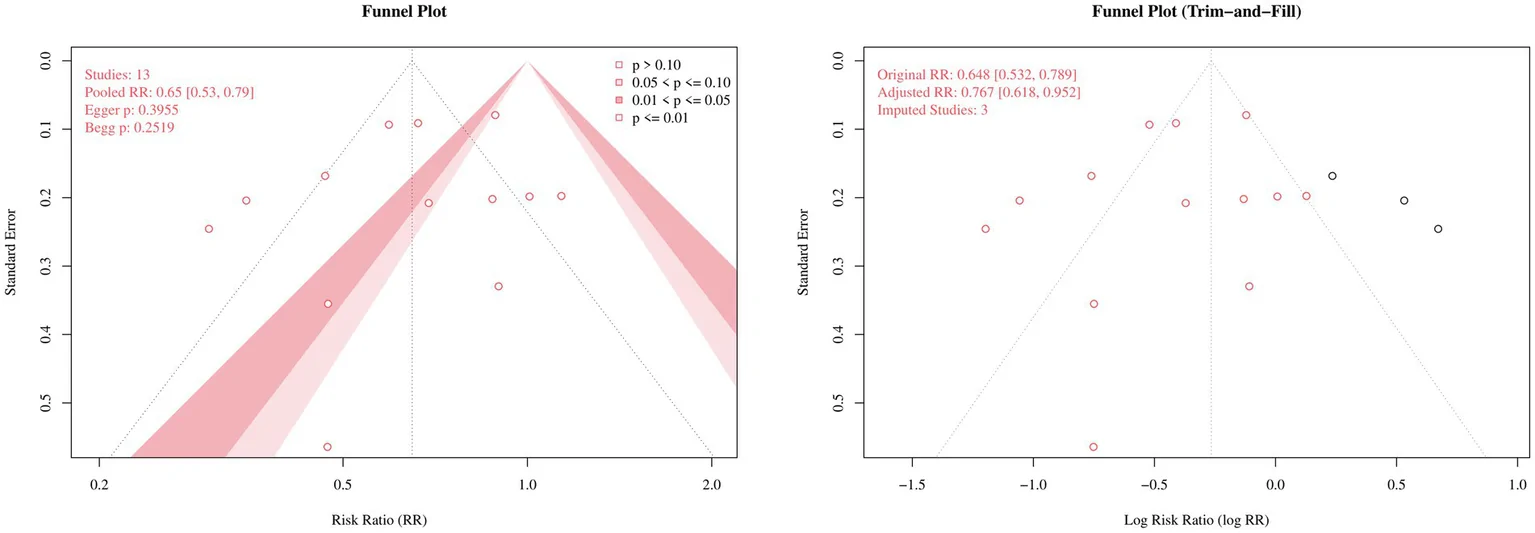
Funnel plot and trim-and-fill sensitivity analysis exploring small-study effects and funnel-plot asymmetry for the overall-injury meta-analysis.

#### GRADE assessment of the certainty of evidence

The certainty of evidence for the primary overall-injury outcome was rated as low after downgrading one level for risk of bias and one level for inconsistency. Risk of bias was downgraded because all included trials raised some concerns, whereas inconsistency was downgraded because substantial between-study heterogeneity was observed (I^2^ = 78%) and the 95% prediction interval (0.33–1.27) included the null effect. No additional downgrades were applied for indirectness, imprecision, or publication bias. Domain-level GRADE judgements and supporting rationale are presented in Supplementary Table S2.

## Discussion

### Principal findings

This systematic review and meta-analysis of 13 unique randomised controlled trials (*n* = 13,675) showed that structured IPPs reduced overall injury risk in female team-sport athletes by approximately one-third, with low-certainty evidence. Although heterogeneity was substantial, most trial estimates favoured IPPs. Leave-one-out, fixed-effect, and exclusion-based sensitivity analyses yielded similar pooled estimates, whereas the trim-and-fill sensitivity analysis attenuated the magnitude of the pooled effect estimate.

When outcomes were considered from broader to more specific injury categories, IPPs reduced the risk of lower-limb, knee, ankle, and ACL injuries, with the most consistent estimate for ACL injury, whereas effects on hip/groin and hamstring injuries remained imprecise because few studies contributed data.

### Interpretation by outcome type

#### Overall injuries

The observed reduction in overall injury risk was broadly compatible with previous meta-analyses conducted in mixed-sex or non-sex-stratified populations (5, 6, 8).

Substantial heterogeneity likely reflects variation in programme design, intervention duration, adherence, sport, and exposure across trials. IPPs should therefore be viewed as a flexible preventive framework rather than a uniform intervention (1, 2). The magnitude of the relative risk reduction should also be interpreted in relation to baseline injury risk, because a substantial relative effect does not necessarily imply an equally large absolute reduction in injuries.

#### Lower-limb and site-specific injuries

Protective effects for knee and ankle injuries were broadly consistent with previous evidence (4, 9), while the ACL estimate was particularly consistent across trials. This may reflect the emphasis of many IPPs on modifiable landing and cutting mechanics associated with ACL injury (11, 40, 41).

#### Context and type: training, match, acute, severe, and non-contact injuries

Protective effects were observed for training-related and acute injuries, whereas effects on match-related, severe, and non-contact injuries remained uncertain. The severe-injury estimate was based on one trial, and the non-contact analysis included only two trials with very high heterogeneity. Because no formal interaction test compared training- and match-related effects, these estimates should not be interpreted as evidence that IPPs differ in effectiveness between settings. Overuse and chronic injuries were not analysed separately.

### Dose, adherence, and other moderators

#### Training dose

Exploratory subgroup estimates suggested greater protection with programmes delivered at least twice weekly and maintained for 4–12 months, although several subgroups contained few trials or substantial residual heterogeneity. Because weekly training dose is derived from session frequency and duration, findings across these related dose variables should not be regarded as independent evidence of separate moderator effects. Weekly training volume showed the clearest pattern, and the continuous meta-regression supported an inverse study-level association between exposure and injury risk. Weekly volume may provide a useful summary of implementation dose because it combines session frequency and duration; however, the available evidence does not establish an optimal or causal threshold.

#### Adherence

Trials with adherence ≥75% showed a larger pooled effect than those with lower adherence, but the continuous meta-regression was not statistically significant. The discrepancy between categorical and continuous analyses may reflect limited power and sensitivity to the selected cut-point; therefore, 75% should not be interpreted as a definitive threshold. A broader multi-sport meta-analysis found a significant continuous association between adherence and injury rates; the non-significant association in the present review may reflect the smaller and more restricted set of female team-sport trials included here (12).

#### Other potential effect modifiers

Sport-type differences were statistically significant, but the non-football and mixed-sport subgroups each contained only two trials. Interactions for age, competitive level, and programme type were not significant, and several subgroups were small or showed substantial residual heterogeneity. These analyses therefore do not establish that effectiveness is determined by a specific sport, age group, competitive level, or programme type.

#### Methodological considerations and limitations

All trials were judged as raising some concerns about risk of bias, and none was judged to be at high risk. Leave-one-out, fixed-effect, and exclusion-based sensitivity analyses yielded broadly similar pooled estimates. Heterogeneity was substantial (I^2^ = 78%), and the prediction interval included 1. Variation in injury definitions, exposure assessment, intervention content, and incomplete reporting of training dose and adherence may have contributed to heterogeneity and reduced the data available for some subgroup and meta-regression analyses. Several secondary outcomes were informed by few trials, resulting in imprecise estimates. In addition, no additional correction for clustering was applied, and the precision of some estimates from cluster-randomised trials may therefore have been overestimated. Dose–response and adherence analyses were based on study-level aggregate data and should not be interpreted as individual-level causal effects. Age group and competitive level overlapped strongly across trials, limiting assessment of their independent moderating effects. Generalisability is also limited because most participants were amateur soccer players. The pooled estimate should therefore be interpreted as an average effect across heterogeneous programmes and settings. Although Egger’s and Begg’s tests were not statistically significant, the limited number of trials restricts our ability to exclude small-study effects. In addition, funnel-plot asymmetry and the attenuation observed in the trim-and-fill sensitivity analysis may reflect genuine between-study heterogeneity, publication-related mechanisms, or both, and should therefore be interpreted cautiously.

## Future directions

Future research should address these limitations and refine implementation in three main areas. First, adequately powered randomised trials are needed that prioritise hip/groin, hamstring, non-contact, and match-related injuries as primary outcomes and include sufficient numbers of adult and elite female athletes. Second, upcoming studies should adopt harmonised injury definitions, systematically measure and report exposure, training dose, and adherence, and present these data in a standardised form to enable more robust dose–response and effect-modifier analyses. Third, embedding IPP evaluations within implementation science frameworks will be essential to understanding how programmes can be scaled, adapted, and sustained in real-world training environments (42).

### Perspective

Exercise-based IPPs appear to reduce injury risk in female team sports, with the most consistent evidence for ACL and ankle injuries. Study-level analyses suggested larger effects with greater weekly training exposure, although evidence for an adherence threshold was less consistent. These findings may inform implementation while highlighting uncertainty for severe, match-related, and non-contact injuries and for adult and elite athletes.

## Conclusion

Exercise-based IPPs appear to reduce overall and lower-limb injury risk in female team-sport athletes, with protective effects also observed for ACL and ankle injuries. Study-level analyses suggested larger effects with greater weekly exposure and in trials with higher categorical adherence, although the continuous adherence meta-regression was not significant. These findings should be interpreted as exploratory implementation signals rather than causal thresholds.

## Data Availability

The original contributions presented in the study are included in the article/Supplementary material, further inquiries can be directed to the corresponding author.
