# Supplementary material for: Effectiveness of injury prevention programmes in female team sports: a systematic review and meta-analysis of hierarchical injury outcomes, training dose–response relationships, and adherence
Source: Front Public Health. 2026 Sep 15;14:1950998. doi: 10.3389/fpubh.2026.1950998 (PMC13619405; doi:10.3389/fpubh.2026.1950998)
Supplement: Supplementary file 1 [file Supplementary_file_1.DOCX]

Supplementary Materials

Supplementary Table S1. Search strategies for all databases.

| **Database** | **Retrieval Strategy** |
| --- | --- |
| **PubMed** | #1 "Athletic Injuries"[Mesh] OR injur*[Title/Abstract] #2 "Female"[Mesh] OR female*[Title/Abstract] OR woman[Title/Abstract] OR women[Title/Abstract] OR girl*[Title/Abstract] #3 soccer[Title/Abstract] OR football[Title/Abstract] OR basketball[Title/Abstract] OR volleyball[Title/Abstract] OR handball[Title/Abstract] OR floorball[Title/Abstract] OR futsal[Title/Abstract] OR rugby[Title/Abstract] OR netball[Title/Abstract] OR lacrosse[Title/Abstract] OR "field hockey"[Title/Abstract] OR "team sport"[Title/Abstract] OR "team sports"[Title/Abstract] #4 "neuromuscular training"[Title/Abstract] OR neuromuscular[Title/Abstract] OR "injury prevention"[Title/Abstract] OR "prevention program"[Title/Abstract] OR "prevention programs"[Title/Abstract] OR "prevention programme"[Title/Abstract] OR "prevention programmes"[Title/Abstract] OR "warm-up"[Title/Abstract] OR warmup[Title/Abstract] OR "strength training"[Title/Abstract] OR "balance training"[Title/Abstract] OR propriocept*[Title/Abstract] OR plyometric*[Title/Abstract] OR "agility training"[Title/Abstract] OR "core stability"[Title/Abstract] OR "preseason conditioning"[Title/Abstract] OR "pre-season conditioning"[Title/Abstract] OR FIFA[Title/Abstract] OR PEP[Title/Abstract] OR "Knee Control"[Title/Abstract] #5 "Randomized Controlled Trial"[Publication Type] OR random*[Title/Abstract] #6 #1 AND #2 AND #3 AND #4 AND #5 |
| **Web of Science** | TS=((injur*) AND (female* OR woman OR women OR girl*) AND (soccer OR football OR basketball OR volleyball OR handball OR floorball OR futsal OR rugby OR netball OR lacrosse OR "field hockey" OR "team sport" OR "team sports") AND ("neuromuscular training" OR neuromuscular OR "injury prevention" OR "prevention program*" OR "prevention programme*" OR "warm-up" OR warmup OR "strength training" OR "balance training" OR propriocept* OR plyometric* OR "agility training" OR "core stability" OR "preseason conditioning" OR "pre-season conditioning" OR FIFA OR PEP OR "Knee Control") AND (random*)) AND DT=(Article) |
| **Embase** | #1 'injury'/exp OR injur*:ti,ab #2 'female'/exp OR female*:ti,ab OR woman:ti,ab OR women:ti,ab OR girl*:ti,ab #3 'soccer'/exp OR 'basketball'/exp OR 'volleyball'/exp OR 'handball'/exp OR soccer:ti,ab OR football:ti,ab OR basketball:ti,ab OR volleyball:ti,ab OR handball:ti,ab OR floorball:ti,ab OR futsal:ti,ab OR rugby:ti,ab OR netball:ti,ab OR lacrosse:ti,ab OR 'field hockey':ti,ab OR 'team sport':ti,ab OR 'team sports':ti,ab #4 'neuromuscular training':ti,ab OR neuromuscular:ti,ab OR 'injury prevention':ti,ab OR 'prevention program':ti,ab OR 'prevention programs':ti,ab OR 'prevention programme':ti,ab OR 'prevention programmes':ti,ab OR 'warm-up':ti,ab OR warmup:ti,ab OR 'strength training':ti,ab OR 'balance training':ti,ab OR propriocept*:ti,ab OR plyometric*:ti,ab OR 'agility training':ti,ab OR 'core stability':ti,ab OR 'preseason conditioning':ti,ab OR 'pre-season conditioning':ti,ab OR FIFA:ti,ab OR PEP:ti,ab OR 'Knee Control':ti,ab #5 'randomized controlled trial'/exp OR random*:ti,ab #6 #1 AND #2 AND #3 AND #4 AND #5 Limit: [article]/lim |
| **Cochrane Library** | #1 injur*  #2 (female* OR woman OR women OR girl*)  #3 (soccer OR football OR basketball OR volleyball OR handball OR floorball OR futsal OR rugby OR netball OR lacrosse OR "field hockey" OR "team sport" OR "team sports")  #4 ("neuromuscular training" OR neuromuscular OR "injury prevention" OR (prevention NEXT program*) OR (prevention NEXT programme*) OR "warm-up" OR warmup OR "strength training" OR "balance training" OR propriocept* OR plyometric* OR "agility training" OR "core stability" OR "preseason conditioning" OR "pre-season conditioning" OR FIFA OR PEP OR "Knee Control")  #5 #1 AND #2 AND #3 AND #4  Limit: Trials |
| **Scopus** | TITLE-ABS-KEY(injur*) AND TITLE-ABS-KEY(female* OR woman OR women OR girl*) AND TITLE-ABS-KEY(soccer OR football OR basketball OR volleyball OR handball OR floorball OR futsal OR rugby OR netball OR lacrosse OR "field hockey" OR "team sport" OR "team sports") AND TITLE-ABS-KEY("neuromuscular training" OR neuromuscular OR "injury prevention" OR "prevention program*" OR "prevention programme*" OR "warm-up" OR warmup OR "strength training" OR "balance training" OR propriocept* OR plyometric* OR "agility training" OR "core stability" OR "preseason conditioning" OR "pre-season conditioning" OR FIFA OR PEP OR "Knee Control") AND TITLE-ABS-KEY(random*) AND (LIMIT-TO(DOCTYPE,"ar")) |
