# Supplementary material for: Effectiveness of injury prevention programmes in female team sports: a systematic review and meta-analysis of hierarchical injury outcomes, training dose–response relationships, and adherence
Source: Front Public Health. 2026 Sep 15;14:1950998. doi: 10.3389/fpubh.2026.1950998 (PMC13619405; doi:10.3389/fpubh.2026.1950998)
Supplement: Supplementary file 2 [file Supplementary_file_2.DOCX]

**Supplementary Table S2. Summary of findings and certainty of evidence (GRADE) for the primary overall-injury outcome.**

| **Outcome** | **Participants / trials** | **Pooled effect, RR (95% CI)** | **Risk of bias** | **Inconsistency** | **Indirectness** | **Imprecision** | **Publication bias** | **Certainty of evidence (GRADE)** |
| --- | --- | --- | --- | --- | --- | --- | --- | --- |
| Overall sports injury | N = 13,675; k = 13 | 0.65 (0.53–0.79) | Seriousᵃ | Seriousᵇ | Not seriousᶜ | Not seriousᵈ | Not seriousᵉ | Low |

GRADE, Grading of Recommendations Assessment, Development and Evaluation; CI, confidence interval; k, number of trials; N, number of participants; RR, risk ratio.

**ᵃ** Downgraded one level for risk of bias because all included trials were judged as raising some concerns in the RoB 2 assessments.

**ᵇ** Downgraded one level for inconsistency because substantial heterogeneity was present (I² = 78%) and the 95% prediction interval (0.33–1.27) included the null effect.

**ᶜ** Not downgraded for indirectness because the included studies directly addressed the prespecified review question. Generalisability is limited by the predominance of football/soccer and amateur athletes.

**ᵈ** Not downgraded for imprecision because the pooled 95% CI (0.53–0.79) did not include the null effect.

**ᵉ** Not downgraded for publication bias. Egger's and Begg's tests were not statistically significant, although their limited power means that small-study effects cannot be excluded.
